# The roles of the gut microbiota–miRNA interaction in the host pathophysiology

**DOI:** 10.1186/s10020-020-00234-7

**Published:** 2020-11-07

**Authors:** Meihong Li, Wei-Dong Chen, Yan-Dong Wang

**Affiliations:** 1 State Key Laboratory of Chemical Resource Engineering, College of Life Science and Technology, Beijing University of Chemical Technology, Beijing, People’s Republic of China; 2 Key Laboratory of Molecular Pathology, School of Basic Medical Science, Inner Mongolia Medical University, Hohhot, Inner Mongolia People’s Republic of China; 3 Key Laboratory of Receptors-Mediated Gene Regulation and Drug Discovery, The People’s Hospital of Hebi, School of Medicine, Henan University, Henan, People’s Republic of China

**Keywords:** Gut microbiota, miRNA, Host pathophysiology

## Abstract

The gut microbiota regulates the biological processes of organisms acting like ‘another’ genome, affecting the health and disease of the host. MicroRNAs, as important physiological regulators, have been found to be involved in health and disease. Recently, the gut microbiota has been reported to affect host health by regulating host miRNAs. For example, *Fusobacterium nucleatum* could aggravate chemoresistance of colorectal cancer by decreasing the expression of miR-18a* and miR-4802. What’s more, miRNAs can shape the gut microbiota composition, ultimately affecting the host's physiology and disease. miR-515-5p and miR-1226-5p could promote the growth of *Fusobacterium nucleatum *(*Fn*) and *Escherichia coli *(*E.coli*), which have been reported to drive colorectal cancer. Here, we will review current findings of the interactions between the gut microbiota and microRNAs and discuss how the gut microbiota–microRNA interactions affect host pathophysiology including intestinal, neurological, cardiovascular, and immune health and diseases.

## Introduction

There are 10^14^ microorganisms in the human gastrointestinal tract, accounting for about 95% of the human microbiome (Friedland and Chapman 2017; Vogt et al. 2017). Many factors contribute to shaping the gut microbiota including environmental factors, diet, disease state, age, and host genetics while the gut microbiota dictates host’s pathophysiology mainly through genes, proteins or metabolites, which play a critical role in the crosstalk between microbiome and host cells (Bäumler and Sperandio 2016; Stilling et al. 2016). The roles of the gut microbiota on the host not only affect intestinal diseases, but also influence other distant organs such as lung, heart and liver (Qin and Wade 2018). Once the balance between the host and the gut microbiota is broken, it will cause the gut microbiota imbalance and eventually contribute to diseases (Mima et al. 2017), such as intestinal (Yu et al. 2017; Rodríguez-Nogales et al. 2017), neurological (Stilling et al. 2015; Bercik et al. 2011), cardiovascular (Wang et al. 2012, 2011), and immune diseases (Kamada et al. 2013; Wilks et al. 2013).

MicroRNAs are small non-coding RNAs, which consist of 22 nucleotides (Rupaimoole and Slack 2017). Mature miRNAs are single-stranded structures and are mostly produced by Dicer enzyme cleaving single-stranded RNA precursors with a hairpin structure of about 70 bases in size (Okamura et al. 2013). Although microRNAs do not encode proteins, they play important roles in various diseases, mainly through silencing the expression of target genes by binding to the 3′ untranslated region (UTR) of the mRNA of target genes (Rupaimoole and Slack 2017). MicroRNAs have been identified as markers of sporadic human colon cancer and active ulcerative colitis in feces and tissues (Ahmed et al. 2009). What’s more, fecal miRNAs can regulate bacterial composition by specifically targeting bacterial genes (Liu et al. 2016), and they can also be used as markers of microbial fluctuations along with gut pathology in the intestine (Moloney et al. 2018). In addition, the gut microbiota has been found to regulate the host gene expression, including miRNAs, primarily through the gut microbiota metabolites, such as lipopolysaccharide (LPS), butyrate, and amyloids (Hu et al. 2015; Peck et al. 2017). Taken together, these studies suggest that miRNAs may interact with the gut microbiota in regulating host pathophysiology.

In this review, we mainly summarize the gut microbiota–miRNA interactions participating in various biological processes of the host pathophysiology. These interactions may provide new research directions for protecting human health in the future.

## The gut microbiota–miRNA interactions in intestinal homeostasis

The gut microbiota is essential in various biological processes of life (Nakatsuet al. 2015; Burns et al. 2015; Feng et al. 2018). By regulating miRNAs, the gut microbes can be more involved in the regulation of genes and intestinal homeostasis of host (Singh et al. 2012). The intestinal epithelial cells (IECs) are the main cells that interact with intestinal bacteria and are involved in maintaining intestinal homeostasis (Peck et al. 2017; Schroeder and Bäckhed 2016). Peck et al. found that miRNA profiles were very different in IEC subtypes, and the difference was related to microbial status. In their study, miR-375 was only sensitive to microbiota from intestinal epithelial stem cells (IESC), and microbiota promoted the proliferation of IESCs by inhibiting miR-375-3p (Peck et al. 2017) (Table 1). Nakata et al. found that commensal bacteria increased miR-21-5p expression level and promoted the permeability of IECs by regulating ARF4 (Nakata et al. 2017) (Table 1). In addition, the gut microbiota participated in the process of colorectal inflammation and colorectal cancer (CRC) by regulating miRNAs. *Lactobacillus fermentum* and *Lactobacillus salivarius,* known as two intestinal probiotics, were reported to increase miR-155 and miR-223 expression, while *L. fermentum* restored expression of miR-150 and miR-143. This regulation enhanced the intestinal barrier function and the homeostasis of the gut microbiota, which alleviated inflammation of dextra sulfate sodium (DSS)-induced mouse colitis (Rodríguez-Nogales et al. 2017) (Table 1). Besides, *Fusobacterium nucleatum* could inhibit miR-18a* and miR-4802 expression, which participated in autophagy-related pathways, possibly resulting in chemoresistance of colorectal cancer. However, how the gut microbiota regulated miRNAs is still unknown. One possible mechanism is that the gut microbiota is responsible for producing different metabolites, which may regulate miRNA functions (Dalmasso et al. 2014). Dalmasso et al. found that colibactin-producing *Escherichia coli* (*pks* + *E. coli*) produced a genotoxic compound colibactin, which induced the expression of miR-20a-5p and finally promoted colon tumor growth through the secretion of growth factors (Dalmasso et al. 2014) (Table 1). The detailed mechanism by which the gut microbiota influences the expression of miRNAs is worthy to be further investigated as it may serve as an attractive therapeutic tool for intestinal diseases.

**Table 1:** Gut microbiota influences miRNAs in host pathophysiology

Pathophysiology	MiRNA	Targets	Cell signaling pathways	References
IESC proliferation	miR-375-3P			(Peck et al. 2017)
IECS permeability	miR-21-5P	PTEN&PDCD4	ARF4	(Nakata et al. 2017)
IBD&CRC	miR-155			(Rodríguez-Nogales et al. 2017)
	miR-233			
	miR-150			
	miR-143			
	miR-18a*	ULK1	TLR4&MYD88	(Yu et al. 2017)
	miR-4802	ATG7	TLR4&MYD88	
	miR-20a-5P	SENP1	P53	(Dalmasso et al. 2014)
	miR-183-5P			(Hoban et al. 2017)
CNS	miR-182-5P			
	miR-206-3P		BDNF signaling	
	miR-294-5P	Brd2&Slit3kr	Kynurenine pathway	(Moloney et al. 2017)
	miR-146a	CFH,TREM2, SHANK3	NF-κB	(Zhao and Lukiw 2018) (Zhao and Lukiw 2018)
Immune system	miR-10a	IL-12/IL23P40	MyD88 pathway	(Xue et al. 2011)
	miR-146b			(Pang et al. 2018)
	miR-29c			
	let-7b		TLR4 signaling	(Guo et al. 2018)
	miR-130a	TNF-α		(Shi et al. 2018)
Cardiovascular disease	miR-10b	ABCG1&ABCA1		(Wang et al. 2012)
	miR-204	Sirt1	Stat3	(Vikram et al. 2016)

**Fig. 1:**
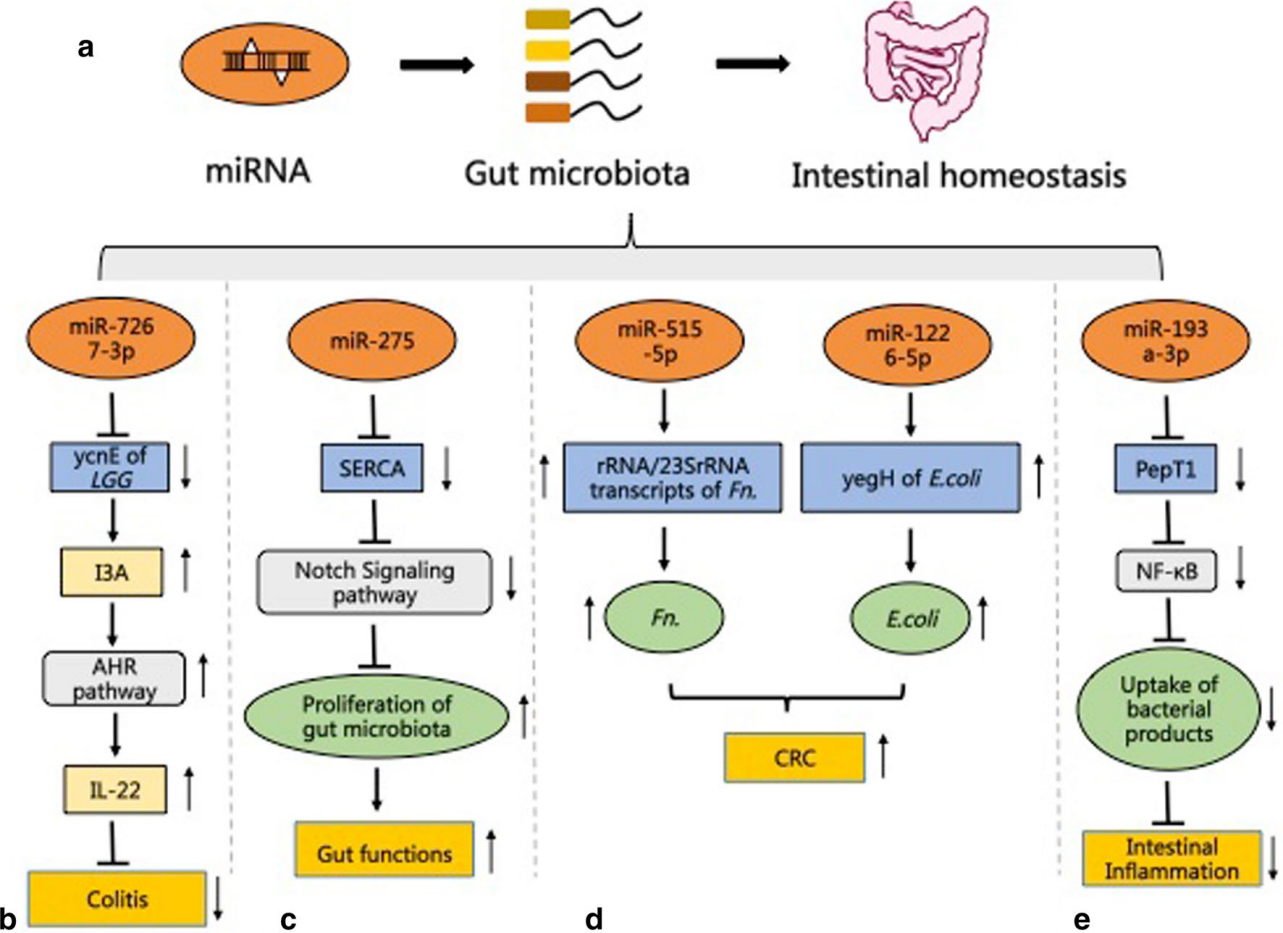
MiRNAs regulate the gut microbiota affecting intestinal homeostasis. **a** MiRNAs regulate the gut microbiota influencing the intestinal homeostasis of the host. **b** The mechanism of miR7267-3p alleviating colitis by repressing ycnE expression of the gut microbiota. **c** MiR-275 protects gut functions in mosquitoes by shaping the gut microbiota. **d** MiRNAs promote the growth of the gut microbiota through the promotion of transcription, affecting the host CRC. **e** MiRNA-193a-3p reduces intestinal inflammation by decreasing the uptake of bacterial products. YcnE, monooxygenase ycnE; LGG, *Lactobacillus rhamnosus*; I3A, indole-3-carboxaldehyde; AHR, aryl hydrocarbon receptor; IL-22, interleukin 22; SERCA, sarco/endoplasmic reticulum Ca^2+^ adenosine triphosphatase; *Fn.*, *Fusobacterium nucleatum*; *E. coli., Escherichia coli*; CRC, colorectal cancer; PepT1, peptide transporter family 1; NF-κB, nuclear factor kappa-B; Pathway diagram key: ┴ inhibition; → induction; ↑ up-regulation; ↓ down-regulation. The figure is referring to the known mechanisms in animal models

Fecal miRNAs can be taken up by the gut microbiota to change the expression of their genes and composition (Liu et al. 2016). Fecal miRNAs are mainly produced by IECs and some Hopx-expressing cells of the host (Liu et al. 2016; Liu and Weiner 2016). The regulation of the gut microbiota by fecal miRNAs is different from traditional miRNA regulation which mainly includes mRNA posttranscriptional repression and results in decreased mRNA translation efficiency (Bartel 2009; Fabian et al. 2010). Bacterial 16S rRNA and ribozyme are also involved in this regulation, and the effects caused by this regulation extend to the promotion of the transcripts (Liu et al. 2016). Liu et al. found that miR-515-5p elevated the proportion of *Fusobacterium nucleatum* 16S rRNA/23S rRNA transcripts, and miR-1226-5p upregulated the level of yegH mRNA in *Escherichia coli*. What’s more, these two miRNAs promoted the growth of *Fusobacterium nucleatum* (*Fn*) and *E.coli,* which has been identified to drive CRC (Liu et al. 2016; Rubinstein et al. 2013) (Fig. 1). Therefore, intestinal fecal miRNAs may affect intestinal homeostasis and gut pathology by also regulating the gut microbiota. miR-21 can aggravate DSS-induced colitis by influencing the gut microbiota (Johnston et al. 2018). The absence of miR-275 could cause intestinal flora reduction in mosquitoes, and the maintenance of miR-275 levels was essential for a variety of intestinal functions in mosquitoes (Zhao et al. 2017) (Fig. 1). Indeed, fecal miRNAs have been identified as independent, non-invasive biomarkers of imbalance at the host-microbe interface, which reflects fluctuations in intestinal microbes and intestinal pathology (Moloney et al. 2018). Ji et al. found that different fecal miRNA expressions in IBD patients affected the progression of IBD by regulation of the growth of some bacteria. The differentially expressed miRNAs in IBD include miR-199a, miR-1226, miR-548a, and miR-515-5p. They affected the growth of pathogen *Fn* and *E. coli* and the probiotic *segmental filamentous bacteria* (SFB), ultimately leading to the occurrence of IBD (Ji et al. 2018). Besides, miRNAs can regulate the uptake of bacterial products to affect the intestinal pathology of the host (Dai et al. 2015). For example, miR-193a-3p was reported to affect the absorption of bacterial products to ameliorate DSS-induced colonic inflammation by targeting PepT1, which helped to absorb bacterial products, and its expression increased in colonic tissues with inflammation (Ayyadurai et al. 2013; Dalmasso et al. 2010) (Fig. 1). Antibiotic treatment could eliminate the effects of miR-193a-3p in DSS-induced enteritis (Dai et al. 2015). Collectively, these results reflect that the gut microbiota–miRNA interactions are plausible in regulating intestinal homeostasis. Attaching importance to this communication is necessary for the treatment of intestinal diseases.

## The gut microbiota–miRNA interactions in central nervous system

The gut microbiota may participate in the regulation of the brain and behavior, especially on normal anxiety and fear behaviors in animals (Neufeld et al. 2011; Clarke et al. 2013; Hoban et al. 2018). The amygdala and prefrontal cortex (PFC) are involved in anxiety disorders and fear responses (Calhoon and Tye 2015). Previous studies have shown that the loss of the gut microbiota throughout life led to transcriptional regulatory changes in the amygdala and PFC (Stilling et al. 2015; Hoban et al. 2016). Also, miRNAs in the amygdala and PFC have been identified to be involved in anxiety and fear-related behavior in mice (Griggs et al. 2013; Dias et al. 2014). A recent report confirmed that the gut microbiota, with the involvement of amygdala and prefrontal cortex miRNAs, participated in the regulation of central nervous system (CNS) (Hoban et al. 2017). Germ free (GF) mice had a large number of abnormally expressed miRNAs in amygdala and PFC. miR-182-5p and miR-183-5p, which have been shown to be associated with stress and fear, were significantly reduced in the amygdala of the GF group and tended to reach normal levels in the germ-free colonized mouse (exGF) group. miR-206-3p, which differently affected anxiety levels in PFC and amygdala by inhibiting the BDNF signaling (Miao et al. 2018), was identified as a target of the gut microbiota (Hoban et al. 2017) (Table 1). Although the detailed mechanisms by which the gut microbiota affected the expression of miRNAs and how miRNAs influence the CNS functions need to be further studied, this discovery could provide a novel way to treat neurological diseases.

The hippocampus is associated with anxiety, depression and cognitive function, and it is receptive to microbiome–gut–brain axis signaling when some neurological function changes in germ-free animals (Clarke et al. 2013; Luczynski et al. 2016; Ogbonnaya et al. 2015). miRNAs in the hippocampus are important participants in gut-brain regulation. A recent study showed that when colonizing GF mice with the gut microbiota from specific pathogen-free (SPF) mice, 7 differentially expressed miRNAs and 139 mRNAs in the hippocampus could be restored (Chen et al. 2017). Similarly, Moloney et al. reported that the gut microbiota influenced mRNA expression patterns in hippocampus by regulating miRNAs, which is critical to hippocampal development. miR-294-5p, involved in the regulation of the kynurenine pathway enzymes, was upregulated in male GF mice and was normalized following colonization. The expression of target genes of miR-294-5p was increased in the kynurenine pathway in male GF mice (Moloney et al. 2017) (Table 1). In addition, miRNAs can regulate the gut microbiota existing in hippocampus, and the regulation participated in host cognitive impairment. Total abdominal irradiation (TAI) exposure is a common means of treating abdominal and pelvic malignancies. It can up-regulate miR-34a-5p expression and change bacterial composition, mediating cognitive dysfunction by targeting Bdnf, which has been reported to participate in many neurodegenerative disorders (Zuccato and Cattaneo 2009). Interestingly, the inhibition of miR-34a-5p by an antagomir could alleviate TAI-mediated cognitive impairment and restore the composition of the gut microbiota (Cui et al. 2017). Therefore, it is reasonable to speculate that the gut microbiota is associated with cognitive impairment, which can be regulated by miR-34a-5p. However, the detailed mechanisms by which the gut microbiota and cognitive impairment changed need more in-depth researches and in vivo experiments.

It has been shown that the gut microbiota dysbiosis affected the formation and development of Alzheimer’s disease (AD), mainly through microbial metabolites, such as amyloids, Lipopolysaccharide (LPS), and short-chain fatty acids (SCFAs) (Sampson et al. 2016; Köhler et al. 2016). miRNA, as an important regulator, was involved in the gut–brain axis related to AD. The gram-negative bacteria *Bacteroides fragilis* and the neurotropic herpes simplex virus-1 (HSV-1) were common in the intestines. They both stimulated the innate immune and neuroinflammatory pathways by activating nuclear factor kappa-B (NF-κB) and inducing microRNA-146a. *B. fragilis* toxins and *B. fragilis* LPS induced up-regulation of NF-κB signaling and the expression of miR-146a in primitive cultured cells derived from human central nervous system (CNS) tissue. At the same time, HSV-1 infection induced the expression of miR-146a through regulating NF-κB and AD-type inflammatory gene signaling in human neuronal-glial (HNG) cells in primary culture (Zhao and Lukiw 2018). It is worth mentioning that the NF-κB-miR-146a signaling pathway, as a part of the innate immune system, appeared to be a proinflammatory factor involved in gut-brain communications, always acting as an Alzheimer's disease inducer (Lukiw 2004; Lukiw et al. 2010; McManus and Heneka 2017). Zhao et al. found that LPS, which is produced by bacteria and is an NF-kB inducer, increased the level of miR-34a and miR-146a. Their target genes, including SH3 and multiple ankyrin repeat domains 3 (SHANK3), triggering receptor expressed on myeloid cells 2 (TREM2), complement factor H (CFH), and tetraspanin 12 (TSPAN12), were closely related to sporadic AD. However, how the secreted toxins from the gut microbiota gradually penetrated the gastrointestinal barrier into the systemic circulation and entered the CNS compartments through the blood–brain barrier is still unknown (Zhao and Lukiw 2018) (Table 1).

## The gut microbiota–miRNA interactions in cardiovascular disease

The gut microbiota–miRNA interactions are involved in the regulation of epithelial dysfunction, which affects the cardiovascular health of the host. Atherosclerosis is the common pathological basis of a variety of cardiovascular and cerebrovascular diseases, and one of the main pathogenic factors is endothelial dysfunction (Rouyer et al. 2019; Thijssen et al. 2019; Hua et al. 2019). Anthocyanins has been reported to extenuate atherosclerosis and promote cholesterol efflux from macrophages (Xia et al. 2006, 2005; Tsuda 2012). The gut microbiota could metabolize cyanidin-3 to 0-β-glucoside (Cy-3-G) to protocatechuic acid (PCA), which is one of the main components of anthocyanins. PCA could inhibit the expression of miR-10b which targeted ABCA1 and ABCG1 in macrophages, resulting in promoting the occurrence of reverse cholesterol transport (RCT), and finally inhibited the occurrence of atherosclerosis (Wang et al. 2012) (Table 1). Besides, the gut microbiota can up-regulate vascular miR-204 via the STAT3 signaling pathway, followed by inhibition of its target gene Sirt1, resulting in impairment of endothelium-dependent vasorelaxation, which is a precursor of atherosclerosis (Davignon 2004). At the same time, high-fat diet-mediated endothelial dysfunction was reversed in germ-free mice or antibiotic-treated mice by down-regulating miR-204 and up-regulating Sirt1 expression. The authors believed that mediator(s) involved in remote communication existed in the systemic circulation, which were related to microbiome-dependent serum factor(s). They demonstrated that short-chain fatty acids such as acetate and butyrate, produced by the gut microbiota, were not involved in the communication between the gut microbiome and the vascular miR-204 (Vikram et al. 2016) (Table 1). The specific mechanism that how microbiome remotely regulated endothelium-dependent vasorelaxation needs to be further investigated as it may bring a new treatment for cardiovascular disease.

## The gut microbiota–miRNA interactions in host immune system

miRNAs can control the differentiation and function of many immune cells, thus they are essential in innate and adaptive immunity (O’Connell et al. 2010; Gantier 2010). It has been shown that specific miRNAs are involved in immune regulation and took part in innate responses to bacterial and viral infections (Bazzoni et al. 2009; Sheedy et al. 2010; Taganov et al. 2006). Abnormally expressed miRNAs are usually characteristic of some immune diseases such as cancer and autoimmune diseases (Pan et al. 2019; Calin and Croce 2006). However, there are few studies on how the microbiota regulates miRNA expression and thus regulates the host's immune homeostasis. Singh et al. found that miRNAs in murine caecum were differently expressed between GF and conventionally reared mice, and some of their potential target genes could encode junctional and mucus layer proteins, which played an important role in immune regulation (Singh et al. 2012). Symbiotic bacteria downregulated the expression of miR-10a in dendritic cells through the TLR-TLR ligand (TLR1/2 bacterial lipopeptides Pam3CSK (*N*-palmitoyl-S-[2,3-bis(palmitoloxy)-(2RS)-propyl]-CysSer-Lys4), TLR4 (LPS), TLR5 (*E. coli* Flagellin FliC), TLR9 (CpG oligodeoxynucleotide), and NOD2 (muramyl dipeptide)) interaction via the MyD88-dependent pathway. This regulation may help to relieve intestinal inflammation and maintain intestinal homeostasis by targeting IL-12/IL23p40, which played a key role in the innate immune response to commensal bacteria (Xue et al. 2011) (Table 1). Besides, adherent invasive *Escherichia coli* (AIEC) could trigger an excessive mucosal immune response associated with Crohn's disease (CD) in wild-type or IL-10 knockout mice by inhibiting the expression of let-7b and thereby caused overexpression of TLR4 (Guo et al. 2018) (Table 1). Therefore, it suggests that the gut microbiota affects the function of immune system by regulating miRNA expression.

In addition, the ability to fight pathogen infection is essential for all living species. The gut microbiota–miRNA interactions are involved in the host's immune response to pathogen infection (Sesto et al. 2014; Brown and Clarke 2017). Archambaud et al. found that microbiota could interfere with the response of microRNAs after *Listeria* infection (Archambaud et al. 2013). Ten miRNAs with high expression in the intestine were analyzed. The expression of miR-194, miR-143, miR-148a, miR-200b, and miR-378 were different between GF mice and conventional mice after *Listeria* infection, providing the evidence that the gut microbiota participated in immune response to bacterial infection by regulating miRNAs (Archambaud et al. 2013). Similarly, improper use of antibiotics could lead to dysregulation of the gut microbiota, which affected the expression of pulmonary miRNAs, such as miR-146b and miR-29c, leading to enhanced amplification of the pulmonary influenza virus and weakening the immune function of host antiviral infections (Pang et al. 2018) (Table 1). Du et al. have confirmed that miRNAs can regulate the state of the gut microbiota, and the regulation participates in host immune response to pathogen infection (Du et al. 2018). miR-146a deficiency was found to defend against *L. monocytogenes* infection by influencing the gut microbiota (Du et al. 2018). The deficiency of miR-146a was conducive to the homeostasis of the gut microbiota. When infected by *Listeria*, the survival of wild-type (WT) mice co-housed with miR-146a knock-out (KO) mice was higher than that of WT mice raised alone (Du et al. 2018). Dennison et al. found that inhibition of aga-miR-305 enhanced Anopheles gambiae's resistance to *P. falciparum* infection and reduced microbiota, which may be related to the regulation of several immune effector genes with the target genes of miR-305 (Dennison et al. 2015). These studies demonstrated that the gut microbiota–miRNA interactions affected host immune system. Thus, these interactions may hold potential for a new effective treatment method in immune diseases.

## Prospects

Growing evidence shows us that homeostasis of the gut microbiota is an essential part of a healthy body. Recently, a new drug named GV-971 treating Alzheimer's disease by targeting the gut microbiota has been launched in China, which opens up a new therapeutic perspective for the treatment of Alzheimer's disease. The medical value of the gut microbiota attracts our attention. Although the above-mentioned reports identified that the gut microbiota–miRNA interactions influence the host pathophysiology, the specific mechanisms by which the gut microbiota–miRNA interactions work in the host’s physiological process are unclear. For example, which genes and pathways does miR-21 regulate to affect the gut microbiota? Furthermore, which signal molecules derived from the gut microbiota participate in the inhibition of miR-10a and regulation of IBD? Which signaling pathways does the gut microbiota impact to increase intestinal inflammation? In addition, which specific bacteria are regulated by miR-275 and how miR-275 specifically affects the intestinal flora of mosquitoes? What’s more, whether there are other miRNAs involved in the regulation of the gut microbiota which improves host cognitive impairment? Does lack of miR-275 also affect intestinal function in humans and mice? More in vivo experiments and studies of molecular mechanisms need to be carried out to confirm the effects of the gut–microbiota–miRNA interactions on host physiological pathology. Overall, deciphering the signals of the gut–microbiota–miRNA interactions will provide new insights into the crosstalk between the gut microbiota and host, and offer a new direction for the study of mechanisms that affect health and disease.

## Conclusion

In this brief review, we summarize the interactions between the gut microbiota and miRNAs, and these roles of the gut microbiota–miRNAs interaction are involved in the pathophysiological process of the host. The study about these interactions may provide new directions for clinical treatment of intestinal, neurological, cardiovascular and immune diseases.

## Data Availability

Not applicable.

## Abbreviations

AD: Alzheimer’s disease
AIEC: Adherent invasive *Escherichia coli*
CD: Crohn's disease
CNS: Central nervous system
CRC: Colorectal cancer
Cy-3-G: Cyanidin-3 to 0-β-glucoside
*E. coli*: *Escherichia coli*
*Fn*: *Fusobacterium nucleatum*
GF: Germ free
HNG: Human neuronal-glial
HSV-1: Herpes simplex virus-1
IECs: Intestinal epithelial cells
IESC: Intestinal epithelial stem cell
LPS: Lipopolysaccharide
PCA: Protocatechuic acid
PFC: Prefrontal cortex
RCT: Reverse cholesterol transport
SFB: Segmental filamentous bacteria
SPF: Specific pathogen-free
TAI: Total abdominal irradiation
IBD: Inflammatory bowel disease
SCFAs: Short-chain fatty acids
NF-κB: Nuclear factor kappa-B
SHANK3: SH3 and multiple ankyrin repeat domains 3
TREM2: Triggering receptor expressed on myeloid cells 2
CFH: Complement factor H
TSPAN12: Tetraspanin 12
